# The impact of civil war on medical training in Sudan after 2 years- a questionnaire-based survey

**DOI:** 10.1186/s12909-026-09542-8

**Published:** 2026-06-02

**Authors:** Kareem Ahmed, Mahdi Abdalmonem, Mohamed E Elamin, Justin C Konje

**Affiliations:** 1 University of Nicosia, Nicosia, Cyprus; 2 University of Khartoum, Khartoum, Sudan; 3 College for Medical Sciences and Technology, Kasr Al Hager, Aswan, Egypt; 4 Feto Maternal Centre, Al Markhiya Street, Doha, Qatar; 5 Department of Obstetrics and Gynecology, Weill Cornell Medicine,(WCM-Q), Doha, Qatar; 6 Department of Health Sciences, University of Leicester, Leicester, UK

**Keywords:** Civil war, Medical education, Online learning, Manpower planning

## Abstract

**Background:**

The break-out of the civil conflict in Sudan in April 2023 has witnessed widespread infrastructural destruction. Most attempts to gauge the impact of this on medical and dental education, have been limited to the first few months of the conflict and concentrated mainly on the impact of the war on students. Our objectives were therefore to determine the impact of the war on students and teachers 2 years into the war and by inference on healthcare manpower planning.

**Material and methods:**

A cross-sectional online questionnaire-based survey of students and teachers from various universities in Sudan between 1st May and 31st July 2025. The survey of students focused on the current status of their educational institutions and the impact of the war on teaching and their plans. That of the teachers in addition to the themes on the students questionnaire also asked about their views on how the war has affected student numbers.

**Results:**

A total of 32 students and 29 teachers from 27 (out a possible 70) institutions completed the questionnaire. 19 (70.4%) institutions had relocated from their permanent sites to other sites within Sudan or overseas - 10 of these to safer parts of Sudan and 9 to another country (Rwanda and Saudi Arabia). Of those that remained opened on their original site (8) face-to-face teaching was suspended in 2, and clinical teaching suspended in 3. Online teaching increased in all the institutions (from <5% to 46.7%). The teachers estimated that the student dropout rate varied from 13-100%. The most suggested reason for drop out by the teachers was financial difficulties (44.8%). 30% of staff had left their jobs and relocated within Sudan for safety reasons or moved to another country.

**Conclusions:**

The civil war in Sudan has caused significant disruption in medical education. This will have lasting consequences on the provision of healthcare in Sudan.

**Clinical trial number:**

Not applicable.

**Supplementary Information:**

The online version contains supplementary material available at 10.1186/s12909-026-09542-8.

## Introduction

Healthcare delivery in any country is influenced by availability of appropriately trained (local and overseas) manpower. Any interruption in training, undoubtedly has serious repercussions on current and future manpower needs of the country. This is more so in low and middle-income countries where the ratio of healthcare provider to patients is much lower. In Sudan for example the ratio of physicians to the population was 2.1/10,000 population in 2021 (significantly lower than the recommended 134/10,000) [1, 2]. Manpower planning to meet healthcare needs of any country are determined by the health of the communities - how stable it is and the prevalence of factors that increase morbidity and mortality. Where there is stability, such planning is an iterative process influenced to an extent by health indicators such as the pattern of diseases and mortality and morbidity. In conflict zones, there is a shift from factors that promote good health and manpower training to a dominance of factors that promote and sustain poor health and thus significantly affect any prospective healthcare manpower planning.

In Sudan where medical training started in 1924, there has been an exponential increase in the number of medical schools and students (both from Sudan and overseas) [3]. As of 2008, there were more than 70 medical schools producing over 5000 graduates yearly, a significant number of who came from other African countries. It was estimated that Sudan had up to 23% of medical schools in Sub-Sahara Africa [4, 5]. Disruption in this setting is therefore likely to have significant consequences not only on health care delivery in Sudan but in the wider region. Several studies have shown that with outbreak of conflict there is often significant disruption in training and furthermore there is deterioration in health driven by the impact of the conflict on displacement of people, famine, and poor living conditions. Tekulu et al. [6] showed that extensive damage to healthcare facilities in the Eastern Tigay Zone led to total collapse of the healthcare system.

The outbreak of the Sudanese Civil War in April 2023 has destabilized the medical education system and the civilian society. This war which quickly swept through the capital to other areas of the country has vastly changed the country’s landscape in every conceivable way. None more so than its Education and Healthcare systems. According to a 2024 report by Doctors Without Borders, up to 80% of people in the capital (Khartoum) have fled for safety [7]. Mahgoub et al. [8] reported that of 58 universities involved in their study (a few months after the onset of the conflict), 58.6% had been vandalized and occupied.

As the conflict intensified and population displacement and damage to infrastructure especially in the capital accelerated, Universities across the country and especially the capital were either temporarily shut down or relocated in contrast to the circumstances at the time of the study by Mahgoub et al. [8]. Even amongst those universities that were able to relocate and adjust so that students could continue their education, most of them faced several challenges. Furthermore, some of the students from the relocated institutions were unable to relocate, for financial reasons, personal safety or otherwise. These problems have persisted more than two years since the start of the conflict. While there have been studies on the wider impact of civil wars/national wars on students, very few have investigated the impact of the wars on students and teachers.

The aims of this study were therefore to survey medical students, some of whom had been displaced by the civil to gain a better understanding of the impact of the conflict on their training and to understand what alternate plans were being made for training. Additionally, we aimed to investigate the impact of the conflict on staff especially with regards to teaching and long-term plans.

## Materials and methods

This was a cross-sectional questionnaire-based survey of students and teachers in Sudanese Universities for the period May - July 2025.

### Survey design

At the outset, we designed a self-administered questionnaire that consisted of 4 sections (Appendix). These sections were (a) demographics (covering the discipline being studied, name and location of institution), (b) status of the educational institution (temporary closed, relocated within or outside Sudan), (c) the impact of the war on studies (on-line teaching, clinical/bed-side teaching) and for the teachers, the impact of the war on them and their colleagues and perceived estimates of the proportion of colleagues and students who might have left the university and reasons for leaving) (d) future plans (continuing with the course, staying in Sudan after completion of studies, moving overseas for postgraduate training or changing their course; and for the teachers - continuing with their current institution, moving to another country and working there or giving up teaching ).

### Questionnaire development

The questionnaires were initially completed (pilot study) by three students and two teachers with whom we had contacts (i.e. conveniently sampled). This was to evaluate the wording, clarity and understanding of the questions to ensure that ambiguity was eliminated. The questionnaires were modified based on the feedback from their responses without altering the structure or content of the questions. The final questionnaires were then shared with two senior colleagues at WCM-Q who reviewed them for content suitability. The questionnaires, which were not subjected to an objective test of internal consistency and reliability were then uploaded onto Google Forms for distribution. The individuals in the pilot stage were included in the final sample analysed.

### Responders and sampling

We recruited students and teachers from the list of universities listed in the World Directory of Medical Schools Database for Sudan. Prior to the war, there were at least 70 institutions. Because of the impact of the war on individuals, it was difficult to access institutional email hence direct recruitment from institutions was not possible. Recruitment was therefore convenient (i.e. we approached easily accessible potential responders) complemented with snowballing (where those who had completed the forms reached out to others). The institutions were not systematically targeted. Responders were identified by personal contacts and through emails and social media. A total of 49 invitations were sent out in the hope that with snowballing we would have a higher response rate (i.e. returned completed questionnaires). An invitation link containing the survey was emailed to each of the contacts who was then encouraged to circulate it to as many potential responders as possible. Each responder gave an informed consent to be part of the survey having been assured that neither their names nor those of their institutions would be included in the published analysis.

### Data collection and analysis

Received completed forms included emails that were used only for the purpose of cross-referencing to ensure that there were no duplicated submissions. Each completed and anonymised (the identity of the responders including their emails and their institutions were removed) questionnaire was then inputted onto a Microsoft Excel database. The database was password protected. We had aimed to recruit a minimum of 60 students and 50 teachers (minimum of 3 per institution) but after an initial flurry of responses, these stopped coming in hence, we stopped the study. Descriptive statistics were generated using GraphPadV.5. These are presented as frequency (numbers) and percentages in Tables and a Pie chart.

## Results

**Fig. 1:**
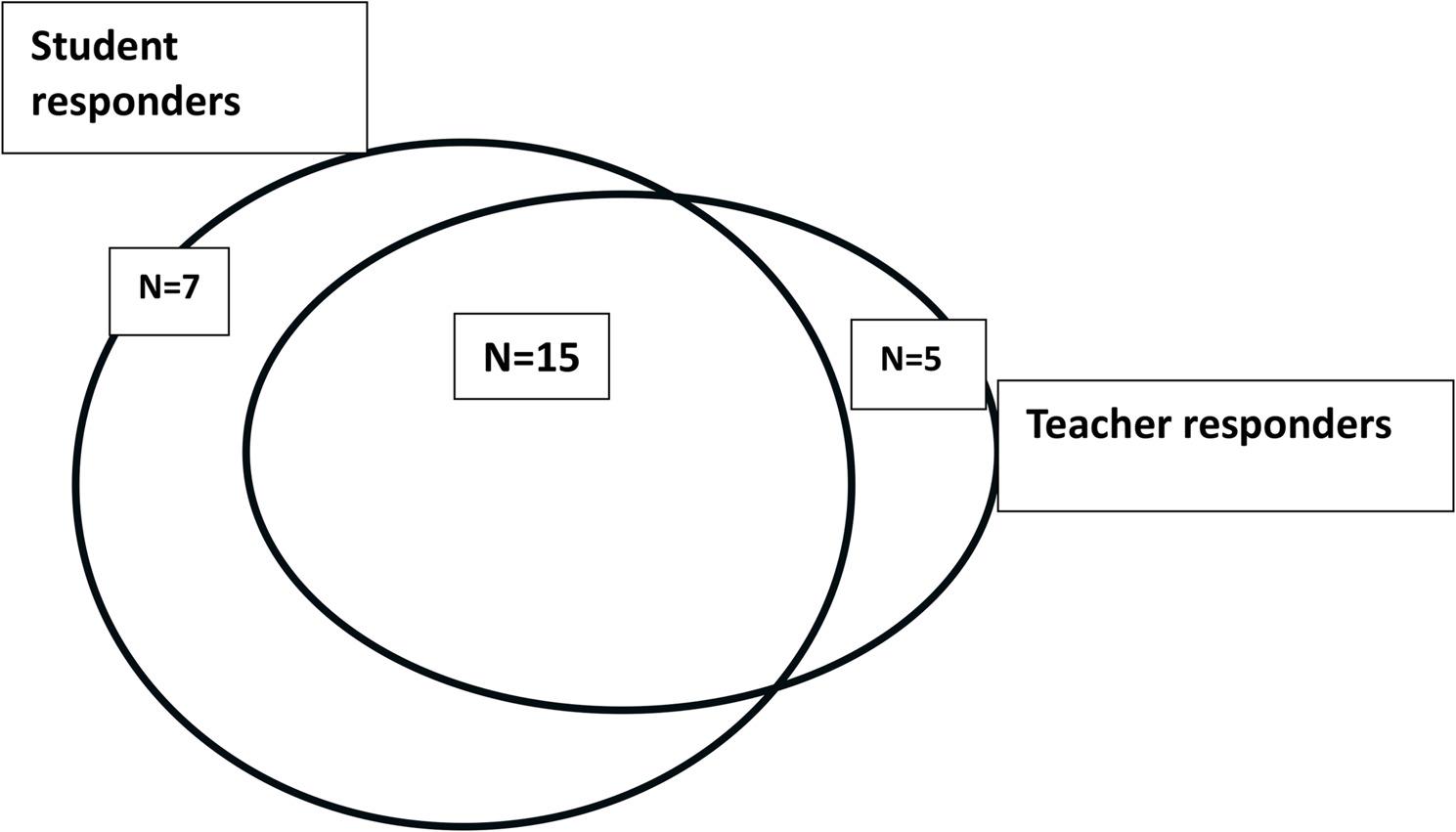
Venn diagram of the overlap of the institutions from which the students and teachers came. Responders (teachers and students) came from the same institution in 15 cases, but students only, came from 7 institutions and teachers only from 5

From the 49 invitations sent out (40 to students and 9 to teachers) a total of 32 responses from students and 29 from teachers were received. These were from a total of 27 institutions out of a possible total of 70 institutions. The students were from 19 institutions and the teachers from 12. Questionnaires were received from both students and teachers of 15 institutions and from the other 12, there was no overlap (7 and 5 from students and teachers respectively). Figure 1 shows the overlap of the institutions from which these completed questionnaires came. Table 1 summarizes the details of the responses received and analyzed. There were 5 student responses from 1 institution, three from 3 and 2 from 3 institutions. There was only one response each from 12 institutions. With the regards to the teachers’ responses, 2 each came from 3 institutions, 5 each from 2 institutions and 1 each from 5 institutions. Twenty-eight (93.3%) of the students were medical, 2 (6.7%) were dental, 1 (3.3%) was a student nurse and 1 (3.3%) did not specify.

**Table 1:** Details of responders and Institution distribution*

Number of responders per institutions	No of institutions from which student responders came (*N* = 19)	No of institutions from which teacher responders came (*N* = 12)
5	1	0
3	3	2
2	3	5
1	12	5

* The students responders came from 19 institutions, and the teachers’ responders from 12 (5, 3 each and 2 each student responders came from 1, 3 and 3 institutions respectively; 3 each and 2 each teacher responders came from 2 and 5 institutions respectively). There was an overlap in some of the institutions (i.e. some of the students and teachers came from the same institutions and in some either only students or teachers completed the survey)

Table 2 shows the distribution of the responders with regards to where their institutions were located prior to the war. Of the 32 responses received from the students, 19 (59.4%) indicated that their institutions were originally based in Khartoum (the city most affected by the civil war), 3 (9.4%) said these were based outside of Khartoum in an area affected by war and 7 (21.9%) in an area outside Khartoum that was not significantly affected by the conflict. The institutions of 21 (72.4%) teachers were based in Khartoum.

**Table 2:** Number of responders against the location of their institution (prior to the war) of the responders

Institution location	Students (*N* = 32)	Teachers (*N* = 29)
Within Khartoum	19 (59.4%)	21 (72.4%)
Outside Khartoum (affected by war)	3 (9.4%0	5 (17.2%)
Outside Khartoum unaffected by war	7 (21.9%)	3 (10.3%)

The state of the institutions at the time of the survey varied widely (Table 3). Nineteen (70.4%) of the schools had relocated from their original sites − 10 (52.6%) relocated within Sudan (mainly Port Sudan), 5 (26.3%) relocated to another country (either Saudi Arabia or Rwanda) as a new institution in those countries while 4 (21.1%) relocated to another country but were hosted by other institutions. The rest (*N* = 8 or 29.6%) remained operational at their original sites. Six (75%) of the 8 institutions that were opened at the time of the survey had been previously shut down for variable periods (4 months − 2 years).

**Table 3:** State of the Institutions at the time of the survey

State of the Institution	Number (27)
Temporarily shut down from their original site and relocated to another • Relocated within Sudan (mainly to Port Sudan) • Relocated to another country as a new institution • Relocated to another country and hosted by another institution	19 (70.4%) • 10 out of 19 (52.5%) • 5 out of 19 (26.3%) • 4 out of 19 (21.2%)
*Remained operational on original site	8 (29.6%)

* These institutions had temporarily shut down for variable periods (4 months to 2 years)

Of the institutions that remained operational on their own campus site (*N* = 8), face-to-face learning was suspended in 25% (*N* = 2) and clinical teaching suspended in 37.5%(*N* = 3). There was an increase in on-line teaching in these institutions (from < 5% to 46.7%). Clinical teaching was suspended in 4 (22%) of the 19 institutions within the country (i.e. relocated and non-relocated). The students who continued schooling reported lack of facilities for clinical teaching in 37.5% of schools and poor access to a broad spectrum of clinical cases in 50% of schools.

Table 4 summarizes the impact of the war on academicians. The most impactful effect was relocation to another part of the country with the same university (*N* = 8; 27.6%) followed by abandonment of teaching (*N* = 7; 24.1%) for safety reasons but would be happy to return once safety was much improved. Nine of them (*N* = 9; 31.0%) had left the universities (i.e. had completely resigned as staff of the University) and had either migrated to other countries or remained in Sudan with no intention of coming back to teaching. Others (*N* = 5; 17.2%) while remaining in the country (most in Port Sudan) refused to teach or were unable to teach. Interestingly the teachers estimated that 30% of their colleagues had left the universities.

**Table 4:** Consequence of the Civil war on the clinical academic (teachers) responders

Consequence of the civil on the teachers	*N* (%) (Total = 29)
Relocated within Sudan with the institution	8 (27.6%)
Abandoned teaching for safety reasons	7 (24.1%)
Left the Universities (resigned as staff)	9 (31.0%)
Refused to teach even after relocating for safety	5 (17.2%)

The estimated student drop-out rate (13.3% − 100%) reported by the staff varied with institution and its location. Whether this drop out was temporary or the students had completely withdrawn from the course was not established. The suggested reasons for the drop out were multiple and included financial (44.8%), safety (48.3%) and inability to move to the new location (36.7%.).

## Discussion

In this cross-sectional questionnaire-based survey we found that 19 (70.4%) of 27 institutions had relocated to either a safer part of the country (10) or to another country (9). Face-to-face teaching was suspended in 25% of institutions still on their original site and on-line teaching increased from < 5% to 46.7% for all the institutions. Clinical teaching had been suspended in approximately 1:5 (20%) of these institutions. While approximately 28% of teachers had relocated with their institutions, 24% had abandoned their roles for fear of safety and another 31% had resigned from the universities and either moved overseas or remained in Sudan with no intention of teaching again. The estimated drop-out rate for students varied from 13 to 100% with most dropping out for financial (44.8%) and safety (48.3%) reasons.

Our finding that about 70 of responders had none or limited access to clinical training sites (defined as relocation with limited access to clinical facilities) closely mirrors the 61.1% reported in a survey of medical students in Sudan by Omer et al. [9]. That cross-sectional study found that 46.1% of responders reported a very high level of disruption in access to medical education and training and 46.9% of universities adopted online classes to ensure continuing education - a figure similar to ours. That same survey found that 16.3% of universities relocated to other countries and a further 5.7% within Sudan. These figures are significantly lower that the 70% in our study. Several factors could explain this difference - firstly at the time of the study of Omer et al. [9] the impact of widespread destruction, and displacement was not at its peak and secondly our numbers are smaller with most of the institutions in our study coming from Khartoum where the greatest devastation on infrastructure and the taking over of institutions by fighters took place.

Our findings confirm that the impact of the civil war on medical education in Sudan is likely to be extensive with long-lasting implications for training and manpower planning. A key factor when considering the impact of war on medical education is the nature of that war. When comparing data from various countries, it is important to consider whether the conflict was civil or nation against nation. Our findings and those of others suggest that a civil war may be more disruptive to medical education than nation-v-nation conflict. The conflict in Sudan was characterised by urban fighting, looting and the conversion of several homes, universities and hospitals to makeshift military bases. In fact, Maghoub et al. [8] in a study shortly after the conflict erupted found that 67.6% of medical schools inside the conflict zones had been converted to military bases. This figure most likely rose with the duration of the conflict.

In previous studies from Liberia [10], Lebanon [11], Croatia [12, 13], Sudan [8] and Ukraine [14] it was shown that conflict has a significant impact on medical education. The students reported a feeling of insecurity, and in some of the cases, had to respond to war by playing the role of healthcare providers despite not completing training. The impact of conflicts was not only on infrastructure and training but also on the mental status of students with a significant number reporting depression [15, 16]). Although this was not one of the questions in our study, we are confident that a significant number of the students and teachers experienced and continue to experience these. Mohammad et al. [17] found that psychological distress (anxiety, depression and post-traumatic stress disorder) was significantly more common in students 8 months into the Sudan war. The relocation of so many institutions in this study likely reflects not only the issues of insecurity but infrastructural destruction associated with looting of equipment; factors which are likely to predispose to psychological disorders.

What is concerning from our study is the number of medical schools that have relocated (70%). A consequence of this is that the students who did not relocate with their institutions either postponed or abandoned their education all together. Those who could afford, likely relocated to other countries or to universities that were still operational. This finding is unique and worrying as long-term, it is likely to have major implications for manpower planning not only for Sudan but for the greater Sub-Sahara regions, as Sudan had up to 23% of the medical schools in this region prior to the conflict.

In some parts of the world where there are conflicts, availability and easy access to broad-band internet means lectures can easily be delivered online. In Sudan where the conflict resulted in a disruption of not only power supply but internet services, delivering this was difficult although some institutions made the effort to do so. That this rose to only 46% means that even those that continued to operate were unable to consistently do so. Even if this was 100%, it would still be inadequate to train healthcare providers, especially as there was complete absence of bed-side training in most cases meaning, doctors completing training would be deficient in clinical skills. Indeed, in a study of 245 students, 8 months into the war, Mohammad et al. [17] reported that many students reported dissatisfaction with online learning.

Financial constraints secondary to the war was the most common reason suggested by the teachers for students dropping out of the university. Our figure of 44.8% was very similar to the 46.9% of students experiencing financial hardship 8 months into the war [17]. In other studies, up to 25% of students dropped out of medical training - a figure significantly higher than the average rate of 11% (range 3–26%) globally [18, 19]). What is equally worrying is the number of those who planned to move overseas - partly because of the desire to further their education (a problem with most low-income countries − 40% in Sub-Sahara Africa and 49% in Ghana [20, 21]).

Mahgoub et a [8]. in their study a few months into the conflict reported that 60.3% of schools had restored some educational processes through measures such as collaboration with other universities both within and outside the country. In our study we did not breakdown the numbers that collaborated with other universities (nationally) after relocating but suspect that the numbers would be much lower as the impact of the conflict had spread more widely within the country compared to earlier when Maghoub et al. undertook their study.

While this study’s primary stated goal was to investigate the effect of the civil war on medical education, we can extrapolate from the findings the potential implications for future manpower planning. The ability to align medical education with a country’s future needs is essential in ensuring improved health outcomes. As quoted by Bosak et al. [22], ‘employing the right number of people with the right qualifications, in the right job and at the right time’ is central to good quality care. Effective planning must consider population growth, epidemiological transition and the current training capacity [23]; all of which have been negatively affected by this conflict. The key questions are how many facilities are likely to reopen at the same capacity as before and how many will be effectively re-staffed since many staff are unlikely to ever return [24].

## Conclusion

Modern medical education in Sudan has a long history, beginning in 1924 with the founding of Gordon Memorial College. The impact of the civil war in Sudan has extended beyond disruption of education and healthcare systems to major destruction of infrastructure and looting of equipment. While healthcare during the war as expected deteriorated, the consequences will be felt much later after the end of the war with much more required to rebuild, equip and staff institutions that would provide training for the manpower needs not only for Sudan but for Sub-Sahara Africa. Furthermore, long-term stability would be critical in reassuring potential staff and students about security and non-recurrence of conflict to encourage return to the country. Whether those institutions that have relocated would come back remains to be seen. 

### Strengths and limitations

The main strength of this cross-sectional study is that it captured data 2 years into the war from both students and teachers. This contemporaneous survey should therefore reflect the impact of the conflict on various aspects of medical education discussed here. Major weaknesses include (a) the limited numbers that were studied with most institutions represented by one student and one teacher (b) the subjective estimates of the rates by individual responders (c) the possibility that the various percentages maybe exaggerated or may in fact be underestimates and (e) that the highly precise percentages we used to present the results could unintentionally convey a degree of statistical certainty not supported by the small sample size. It is only from large studies that the true impact of the ongoing civil conflict in Sudan can be understood and maybe quantified. Finally, the questionnaire employed did not undergo objective validation which may limit interpretation of resulting data.

## Supplementary Information

Supplementary Material 1.

## Data Availability

The datasets analyzed during the current study are available from the corresponding author upon reasonable request.
