# Supplementary material for: The impact of civil war on medical training in Sudan after 2 years- a questionnaire-based survey
Source: BMC Med Educ. 2026 Jun 2;26:1255. doi: 10.1186/s12909-026-09542-8 (PMC13445928; doi:10.1186/s12909-026-09542-8)
Supplement: Supplementary file 1 [file 12909_2026_9542_MOESM1_ESM.docx]

**Impact of Civil War in Sudan on Medical Training and Implications for Manpower Planning**

***Questionnaire for Students***

The civil war in Sudan which started in April 2023, has significantly affected every aspect of the society with a lasting negative impact on families and the society as a whole. This impact is felt much more in education especially medical education with forced closures of schools, and teaching hospitals and displacement students and teachers. This war is now in its third year and just how much this impact has been at this stage has not been investigated. In this survey we wish to find out the impact of the civil war on various aspects of medical education and to then extrapolate the long-term implications for manpower planning for the country. To objectively do this we invite you to complete this questionnaire on various aspects of your education and teaching. This is an anonymous questionnaire that should take you less than 10 minutes to complete. Your personal information will not be disclosed in the analysis. We would be grateful if you could forward the questionnaire to other students to complete and send to the email address above. Thank you for taking part in this important survey.

As an anonymous survey, your email address will be saved with the form to ensure that there is no duplicate submission. It will however not be used or made available in the analysis. For each question, tick your chosen option (s), and where prompted add any narrative that is appropriate. Submit your completed questionnaire to the email account below:

**jckonje@gmail.com** [Switch account](https://accounts.google.com/AccountChooser?continue=https://docs.google.com/forms/d/e/1FAIpQLSczmInirExb59yc0IW7fDoXGa1NZFsyBVoD6rBrE8uRVTW93A/viewform?usp%3Dsend_form&service=wise)

**Questions for Sudan Medical Schools - this is an anonymised questionnaire**

**A. Demographic**

a) Email address:
b) What discipline are you studying f
   I. Medicine
   II. Dentistry
   III. Nursing
   IV. Pharmacy
   V. Physiotherapy
   VI. Other - specify ..............................................
b) Before the war, where was the school located (i.e. in relationship to Khartoum)
   I. Khartoum
   II. Outside Khartoum but in a war region
   III. Outside Khartoum but in a area not affected by the

**B. What is the current situation with your Institution (University)**

a. Continuing at the same site as before (home of the institution)
b. Relocated to within Sudan
c. Relocated to another country - name country...........................................
   i. Running as a stand-alone university
   ii. Hosted by another university

**C. What has been the impact of the war on status of your studies?**

a) Continuing with my studies as normal (same institution and same venue)
b) Suspended my studies
c) Temporarily suspended as I did not want relocate
d) Have financial problems, so could not relocate
e) Suspension of clinical teaching/significant reduction
f) Withdrawn from the course altogether (i.e. will not do it again even after the war)
g) How has teaching been affected:
   i. Face-to-face
   ii. On-line
   iii. Both
h) How has your bed-side clinical teaching been affected?
   i. Not affected
   ii. Stopped completely
   iii. Significantly reduced. By what percentage.?.....%
   iv. Limited spectrum of clinical cases for teaching

**D: What are your plans for the future**

a. Continue with postgraduate studies in Sudan
b. Move to another country and continue my studies or work there
c. No plans to work in Sudan
d. Don’t know

**Impact of Civil War in Sudan on Medical Training and Implications for Manpower Planning**

***Questionnaire for the Teachers***

The civil war in Sudan which started in April 2023, has significantly affected every aspect of the society with a lasting negative impact on families and the society as a whole. This impact is felt much more in education especially medical education with forced closures of schools, and teaching hospitals and displacement students and teachers. This war is now in its third year and just how much this impact has been at this stage has not been investigated. In this survey we wish to find out the impact of the civil war on various aspects of medical education and to then extrapolate the long-term implications for manpower planning for the country. To objectively do this we invite you to complete this questionnaire on teaching and your perceived impact of the war on students. This is an anonymous questionnaire that should take you less than 10 minutes to complete. Your personal information will not be disclosed in the analysis. We would be grateful if you could forward the questionnaire to other teachers to complete and send to the email address above. Thank you for taking part in this important survey.

As an anonymous survey, your email address will be saved with the form to ensure that there is no duplicate submission. It will however not be used or made available in the analysis. For each question, tick your chosen option (s), and where prompted add any narrative that is appropriate. Submit your completed questionnaire to the email account below:

**jckonje@gmail.com** [Switch account](https://accounts.google.com/AccountChooser?continue=https://docs.google.com/forms/d/e/1FAIpQLSczmInirExb59yc0IW7fDoXGa1NZFsyBVoD6rBrE8uRVTW93A/viewform?usp%3Dsend_form&service=wise)

**Questions for Sudan Medical Schools - this is an anonymised questionnaire**

**A. Demographic**

a. Email address:
b. Before the war, where was your school located (i.e. in relationship to Khartoum)
   i. Khartoum
   ii. Outside Khartoum but in a war region
   iii. Outside Khartoum but in a area not affected by the

**B. What is the current situation with your Institution (University)**

a) Continuing at the same site as before (home of the institution)
b) Relocated to within Sudan
c) Relocated to another country - name country...........................................
I. Running as a stand-alone university
II. Hosted by another university

**C. What has been the impact of the war on you and your colleagues?**

a) Relocated with the university
   I. To another pat of Sudan
   II. To another country
b) Left teaching temporarily
c) Left teaching permanently with no plans to return
d) What proportion of your colleagues do you think have left or plan to leave teaching in this university:?.........%
e) What proportion of students do you estimate have withdrawn from studies (if you know) .......%
f) Why do you think are the reasons for the withdrawal? (give as many reasons as possible) ..............................................................................................................................................................................................................................................................................................................................

**D: What are your plans for the future**

a. Continue working with my current university
b. Move to another country and work there
c. Give up teaching completely
d. Do not know
